# Exposure to Kalach, a Glyphosate-Based Herbicide, During Pregnancy and Lactation Induces Hypothyroidism and Bone Disorders in Rat Offspring

**DOI:** 10.3390/toxics13090752

**Published:** 2025-09-04

**Authors:** Latifa Hamdaoui, Hafedh El Feki, Marwa Ben Amor, Hassane Oudadesse, Mohamed Atwan, Ahmed Mohajja Alshammari, Faten Brahmi, Hmed Ben-Nasr, Riadh Badraoui, Tarek Rebai

**Affiliations:** 1 Laboratory of Histophysiology of Developmental and Induced Pathologies (LR19ES12), Faculty of Medicine of Sfax, University of Sfax, Sfax 3029, Tunisia; benamor.ayadi.marwa@gmail.com (M.B.A.); tarekrebaifmsf@gmail.com (T.R.); 2 Laboratory of Materials and Environmental Sciences, Faculty of Sciences of Sfax, B.P. 1171, Sfax 3000, Tunisia; hafed.elfeki@fss.usf.tn; 3 CNRS, Compus Beaulieu, Rennes University, 35000 Rennes, France; hassane.oudadesse@univ-rennes1.fr; 4 Department of Biology, University of Ha’il, Ha’il 55212, Saudi Arabia; mohammad.atwan@yahoo.com (M.A.); dr.mohajja@gmail.com (A.M.A.); f.brahmi@uoh.edu.sa (F.B.); riadh.badraoui@fmt.utm.tn (R.B.); 5 Research Unit UR 12 ES 13, Laboratory of Pharmacology Faculty of Medicine of Sfax, University of Sfax, Sfax 3029, Tunisia; hmedbnasr@gmail.com; 6 Laboratory of Histology-Cytology, Tunis Faculty of Medicine, Tunis El-Manar University, La Rabta-Tunis 1007, Tunisia

**Keywords:** kalach, bone, hypothyroidism, molecular interactions, computational study, in silico

## Abstract

Kalach (KL) is a glyphosate (G)-based herbicide extensively used in agricultural and urban areas in Tunisia. It has been reported that G crosses the placenta in pregnant rats, potentially disrupting organ function in offspring. The present study examined the effects of prenatal and lactational exposure to KL on thyroid function, bone integrity, and phosphocalcic homeostasis in rat offspring. Pregnant rats were divided into two groups, group A (control group) and group B, exposed to KL (each mother rat received 0.07 mL of KL diluted in 1 mL of water by gavage). On postnatal day 14, plasma samples were analyzed for thyroid hormones, calcium, and phosphorus. Histology and immunohistochemical study of bone and thyroid, Fourier-transform infrared (FTIR) spectroscopy, X-ray diffraction (XRD), and scanning electron microscopy assessed alterations. Additionally, we complemented the in vivo study with an in silico study. We found that KL induced hypothyroidism, necrosis in thyroid tissue, and phosphocalcic imbalance, leading to skeletal abnormalities. Structural and mineralization defects in bone were confirmed by FTIR and XRD analysis. The in silico study revealed that G bids to growth hormone receptors and thyroglobulin with good affinity, corroborating the in vivo findings. In conclusion, KL may interfere with bone tissue, growth hormone receptors, and thyroglobulin, impair hypothyroidism, and function as an endocrine disruptor exposure. Consequently, KL induces disorganization of the femoral growth plate.

## 1. Introduction

For many years, the global use of pesticides has significantly increased, particularly in agriculture, where they are applied to improve crop yield and quality while maximizing economic returns [1]. Among these chemicals, glyphosate-based herbicides (GBHs) are extensively used across various crops to combat pests in numerous countries [2]. In Tunisia, one of the most commonly applied GBHs is KL [3].

Glyphosate (G) and its primary metabolite aminomethylphosphonic acid (AMPA) have become a global concern due to their potential to contaminate both surface and groundwater, posing serious threats to biodiversity and public health [4,5]. A recent review highlighted that countries along the southern Mediterranean coast are likely to face rising levels of water contamination linked to the use of GBHs. Strikingly, AMPA and glyphosate were detected in 100% and 94% of analyzed water samples, respectively [6].

Fréville et al. in 2023 examines the key toxicokinetic parameters of G and AMPA following GBH administration in chickens. The results indicated that both compounds are rapidly absorbed and eliminated when ingested. Plasma concentrations are nearly undetectable 48 h post-ingestion, and their low steady-state volume of distribution suggests limited dispersion into peripheral tissues. Since co-formulants may be more toxic than G itself, evaluating their toxicokinetic profiles could be valuable. However, the lack of detailed information on the exact composition of most GBHs complicates such analyses. Additionally, experimental designs incorporating different ages and sexes would be beneficial, given that most fatal poisoning cases occur in older men [7,8].

Therefore, the intensive use of pesticides may pose severe risks to human health, particularly for pregnant women, newborns, and breastfed children [9]. In pregnant rats, lactational exposure to GBHs has been shown to allow G to cross the placental barrier, leading to alterations in brain enzyme activity in both mothers and offspring [10,11,12]. Several studies have detected G in breast milk samples from mothers, highlighting potential risks for breastfed children [13,14,15]. Specific data on G residues in human breast milk contextualized the resulting infant exposure. Briefly, a Brazilian study conducted during peak agricultural application and using ELISA reported a mean of 1.45 µg/L (67/67 positive samples). Assuming ~152.6 mL/kg/day milk intake, this equates to ~0.22 µg/kg/day, i.e., ~0.04% of the EFSA ADI (0.5 mg/kg/day) [16].

It has been reported that G and GHBs affect bone metabolism, raising concern about their impact on bone remodeling [17,18]. Bone remodeling is the lifelong process by which old or damaged bone tissue is resorbed by osteoclasts and replaced with new bone formed by osteoblasts, ensuring bone strength, mineral homeostasis, and adaptation to mechanical stress. It comprises two phases: bone formation and resorption. The balance between the two phases is crucial for sustaining bone mass and systemic mineral homeostasis [19]. This physiological mechanism is regulated through a complex interplay of systemic factors, such as hormones, and local mediators, including various cytokines and growth factors [20]. Calcium and phosphorus plays a crucial role in bone development in the prenatal period, as it is essential for skeletal growth, mineralization, and overall bone strength [21,22]. In addition, thyroid hormones (THs) have a key role in the control of bone homeostasis and are fundamental for normal endochondral ossification, regulating bone mass maintenance, linear growth, and fracture healing [23,24]. The infant thyroid gland is particularly vulnerable to such endocrine-disrupting chemicals, given its essential role in growth, metabolism, and skeletal development [25]. Thus, both thyroid hormone status imbalance and disruptions in calcium homeostasis can consequently indirectly affect bone structure [17,18].

Based on the available literature and our previous findings, GBHs induced hypothyroidism, bone disturbances and deficiency of hormones responsible for phospocalcic metabolism, such as vitamin D (Vit D) and parathyroid hormone (PTH) [17,26,27]. Moreover, our previous study showed that KL induced estrogen deficiency and may trigger pro-oxidant events in rat bone, as evidenced by the increased malondialdehyde content observed in KL-treated rats compared to controls. We therefore hypothesized that KL exerts toxicological effects, including oxidative stress, during bone healing [17]. Numerous studies have shown that estrogen deficiency is closely related to oxidative stress [28,29,30]. Because of the effect of reactive oxygen species (ROS) on mesangial and endothelial cells, changes in both morphological structure and function of the glomeruli were detected [31]. Therefore, it is scientifically reasonable to propose that the observed post-gestational and lactational exposure to GBHs may arise from a dual mechanistic pathway.

Furthermore, despite numerous studies on GBH toxicity in various tissues in pups at the prenatal and lactational period [32,33,34,35], the impact of GBH exposure on bone remodeling, the thyroid gland, mineral composition, and calcium–phosphate balance during the prenatal and lactational period remains poorly understood.

The present study aimed to evaluate the toxic effect of KL exposure during pregnancy and lactation on thyroid function, bone tissue integrity, and calcium–phosphate homeostasis in rat offspring. Additionally, we investigated the molecular interactions of KL with the key metabolic receptors growth hormone (GH) and thyroglobulin using an in silico computational approach.

## 2. Materials and Methods

### 2.1. KL Solution: Preparation Method

The lethal dose (LD_50_) of KL by gavage was 1260 mg of BW (20). KL, G-based herbicide, was purchased from Bioprotection. This herbicide was made available by Monsanto Europe. In this study, we employed the commercial herbicide formulation KL, comprising 360 g/L of glyphosate (G) as the active compound. The molecular structure of G is shown in our previous study [18]. KL contains isopropylamine salt of n-phosphonomethylglycine, (41.5%), water (43%), and adjuvants or surfactant (15.5%).

### 2.2. Animals

Adult female and male Wistar rats (7 weeks old) were procured from the Central Pharmacy (Industrial Society of Pharmaceutics, Tunis, Tunisia). The animals were housed in conditions with an ambient temperature of 23 °C, minimum humidity of 40%, and a 12 h light and 12 h dark cycle. The rats were provided with a commercial diet (bought from SICO, Sfax in Tunisia) and had access to tap water ad libitum. Following a one-week acclimatization period to the laboratory conditions, female and male rats were paired together. Daily monitoring of pregnancy in female rats involved examination for the presence of a vaginal plug, serving as an indicator for the commencement of pregnancy on day zero.

### 2.3. Experimental Procedure

On day 1 of the pregnancy, the pregnant rats (*n* = 12) were equally divided into two groups of six individuals:

❖ Group A (*n* = 6): Composed of six rats, which served as a control, treated in the same way with deionized water (1 mL) for gestation and lactation periods and received a standard diet.
❖ Group B (*n* = 6): Served as a treated group with dose 1. This dose was 1/10 of DL 50 of KL containing 126 mg. Each rat received 0.07 mL of KL dissolved in 1 mL of water by gavage for gestation and lactation periods.

From the first day of pregnancy prior to the onset of fetal thyroid function until day 14 of lactation, group B received treatment with the commercial herbicide KL. Each pregnant rat was housed individually to allow for spontaneous delivery approximately three weeks after mating. At birth, litter sizes were standardized to eight pups per dam (preferably four males and four females), a procedure known to optimize lactation performance [36]. The day of birth was designated as postnatal day one. Eight pups per litter were randomly selected for euthanasia and subsequent analysis.

All experimental procedures were conducted in accordance with the guidelines of the Regional Ethics Committee for Animal Experimentation at the Faculty of Medicine of Sfax (CEREAS) under approval protocol CEREAS-AS4625/19810.

### 2.4. Euthanasia

At postnatal day 14, following ether anesthesia, blood samples were collected from the brachial artery of the rat pups into heparinized tubes and centrifuged at 2200× *g* for 15 min. Thyroid glands and femora were carefully harvested. All blood and organ samples were stored at −80 °C for subsequent biochemical, mineral, and histological analyses, as described in [37].

### 2.5. Determination of Morphological Parameters

Throughout the experimental period, the body weights of both dams and offspring were regularly recorded (*n* = 8). After euthanasia, femora (right or left) from the pups were excised with care to assess their length and weight (*n* = 8) [38].

### 2.6. Sample Preparation

Blood samples were collected from the brachial artery of the pups. Plasma was separated by centrifugation at 3000× *g* for 15 min and stored at −20 °C. Femoral samples were also harvested and preserved at −20 °C for subsequent biochemical and mineral analyses.

### 2.7. Determination of Hormonal Status

A total of eight plasma samples for each group (eight samples for pups from group A and eight samples for pups from group B) were randomly selected for the determination of free thyroxine (FT4) and triiodothyronine (FT3). We using radioimmunoassay, and the results were expressed in pmol/L. Commercial kits from Immunotech, France, with references 1363 (FT4) and 1579 (FT3), were utilized for these measurements.

### 2.8. Metabolic Balance

Levels of calcium and phosphorus in both plasma and bone were assessed using colorimetric techniques with the Cobas 6000 system from Roche^®^. The results are expressed in milligrams per liter.

### 2.9. Histological Analysis of the Thyroid Gland

Six thyroid samples were collected for each group, immediately fixed in 10% neutral buffered formalin, and subsequently embedded in paraffin. Sections of 4 μm thickness were prepared, mounted on glass slides, stained with hematoxylin and eosin (H&E), and examined under a light microscope. For each experimental group, at least four thyroids were randomly selected for microscopic evaluation.

### 2.10. Immunohistochemical Evaluation of Thyroid Cell Function

Paraffin-embedded thyroid sections were dewaxed using standard protocols and subjected to heat-induced antigen retrieval. To block endogenous peroxidase activity, the sections were treated with 3% hydrogen peroxide in distilled water at room temperature. Following this, the sections were incubated for 1 h at room temperature with a blocking solution, then overnight at 4 °C with the primary antibody. The primary antibody used was rabbit monoclonal anti-thyroglobulin (CliniSciences, AC-0220A). Antibody binding was visualized using a streptavidin–peroxidase Histostain-SP kit, with positive staining indicated by brown–yellow coloration.

### 2.11. Femur Histological Analysis

Femoral samples of pups for each group (six samples for pups in group A and six samples for pups in group B) were immediately fixed in BB (ethanol 95°, 40% of formaldehyde, and water H_2_O). After 48 h, the samples were decalcified with 10% nitric acid, dehydrated, and embedded in paraffin blocks. These blocks were sectioned at 4 μm and later stained with H&E for histological examination.

### 2.12. Histological Score Analysis

Histological scores of the thyroid and femur tissues from female rats were assessed using ImageJ software (version 1.48) following the method described by Strissel et al. [39].

### 2.13. Infrared Spectroscopic (FTIR) Analysis of Bone Samples

The FTIR technique was employed to identify the chemical composition of KL and the bonds between atoms. Bone samples were pulverized using a colloid mill cooled with liquid nitrogen (Retsch MM 200), followed by vacuum freeze-drying using a MAXI dry lyo system (Heto). For FTIR analysis, pellets were prepared by thoroughly mixing the dried bone powder with potassium bromide (KBr) at a ratio of 1 mg of sample to 100 mg of KBr under vacuum conditions. FTIR spectra were recorded using a Nicolet Magna-IR 550 spectrometer (Madison, WI, USA) [40]. FTIR spectra were acquired using a Bomem MB157 spectrometer (purged with dry air) over a spectral range of 4000–400 cm^−1^. Each spectrum was obtained by averaging 400 interferograms at a resolution of 4 cm^−1^.

### 2.14. X-Ray Diffraction (XRD) Analysis

X-ray diffraction patterns of the powdered bone samples were recorded using a Bruker D8 diffractometer controlled by an IBM-compatible PC. The instrument was operated at 40 kV and 30 mA, with data collected over a 2θ range of 10° to 70°. This analysis was performed to characterize the crystalline structure of the bone specimens.

### 2.15. Scanning Electron Microscopy (SEM)

Scanning electron microscopy (SEM; JEOL JSM-5100) was conducted on the surgically treated femora of the rat pups and analyzed at 20 kV. In summary, the femurs were longitudinally cut (epiphysis) and prepared as per Atwa et al. [41]. This analysis was conducted at the Laboratory of Solid-State Chemistry and Materials at the University of Rennes 1.

### 2.16. Molecular Interactions and Computational Analyses

The effects of KL were confirmed by in silico molecular docking and interaction assays. The active sites of four receptors were targeting estrogen receptor α (ER α), androgen receptor α (ARα), growth hormone receptor (GH), and thyroglobulin. The tridimensional structure of each macromolecule and G was obtained from the RCSB and PubChem websites, respectively. The docking approach was based on the CHARMm force field after processing the receptors. KL was assessed for its ability to interact with the targeted proteins after removing water molecules and adding the missing polar hydrogens and Kollman charges as previously reported [42]. The binding affinity and hydrogen bond calculations were conducted as previously reported [43,44,45,46]. These receptors were selected because they are commonly disrupted following exposure to several chemicals, including pesticides and endocrine disruptors [28,47,48].

### 2.17. Statistical Analysis

Statistical analyses were conducted using SPSS software (version 26 for Windows; SPSS Inc., Chicago, IL, USA). Quantitative parametric data are expressed as means ± standard deviation (SD) and were compared using Student’s *t*-test. A *p*-value of less than 0.05 was considered statistically significant.

## 3. Results

### 3.1. Growth and Feeding: Effects of KL on Body and Femur Length

Body weight, femur length, and weight of femora of offspring were decreased by 15%, 8%, and 4%, respectively, in pups in group B compared to those of group A (*p* ≤ 0.05, *p* ≤ 0.01) (Table 1), so the dose of KL administered to pups born in group B provoked growth retardation.

### 3.2. Effects of KL on Biochemical Markers in the Bone of Offspring

Table 2 shows biochemical variables that indicated bone injury in offspring in group B. The plasma levels of calcium and phosphorus were increased by 28% and 15% (*p* ≤ 0.05, *p* ≤ 0.01), respectively, in offspring in group B compared to offspring in group A. In addition, femur bone of young rats from group B demonstrated a non-significant decrease in calcium, but a significant decrease in phosphorus by 12% and 15% (*p* ≤ 0.01), respectively, compared to group A (Table 2).

### 3.3. Hormonal Thyroid Variation of Offspring

Levels of TH in plasma, specifically free T4 (FT4) and free T3 (FT3), were significantly reduced by 21% and 20%, respectively (*p* ≤ 0.01), in pups in group B compared to the pups in group A (Table 2).

### 3.4. Histological Study of Thyroid Gland

**Figure 1:**
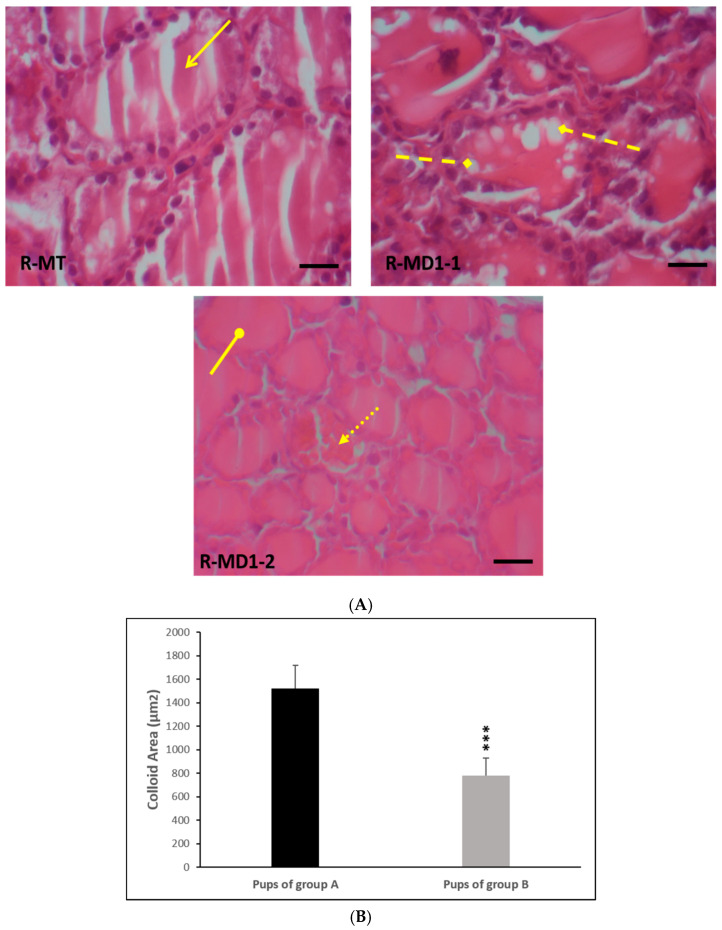
(**A**) Histological thyroid sections of 14-day-old rats using H&E staining. Group A (R-MT1) and pups in group B breastfed by mothers from day 1 of pregnancy until day 14 (R-MD1-1 and R-MD1-2). (**B**) Histomorphometric analysis of colloid area (µm^2^). H&E (200X and 400X). Values are expressed as means ± SD. Significant differences between pups in group A and group B: *** *p* ≤ 0.001. 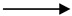: Normal thyroid follicles; 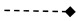: follicular cell with vacuolation; 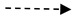: necrosis; 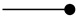: closed follicular cell.

Thyroid glands of the pups in group A displayed normal characteristics (Figure 1A: R-MT). However, biochemical perturbations observed in young rats in group B were confirmed by the histological study of thyroid glands. We noted a necrosis follicular cell, an increase in closed follicular cell number, and a decrease in colloid volume (Figure 1A: R-MD1-1 and R-MD1-2). Histomorphometric analysis showed a decrease in colloid area by 48% (*p ≤* 0.001) in pups in group B compared with pups in group A (Figure 1B).

### 3.5. Immunohistochemical Findings in Thyroid Sections of 14-Day-Old Rats

**Figure 2:**
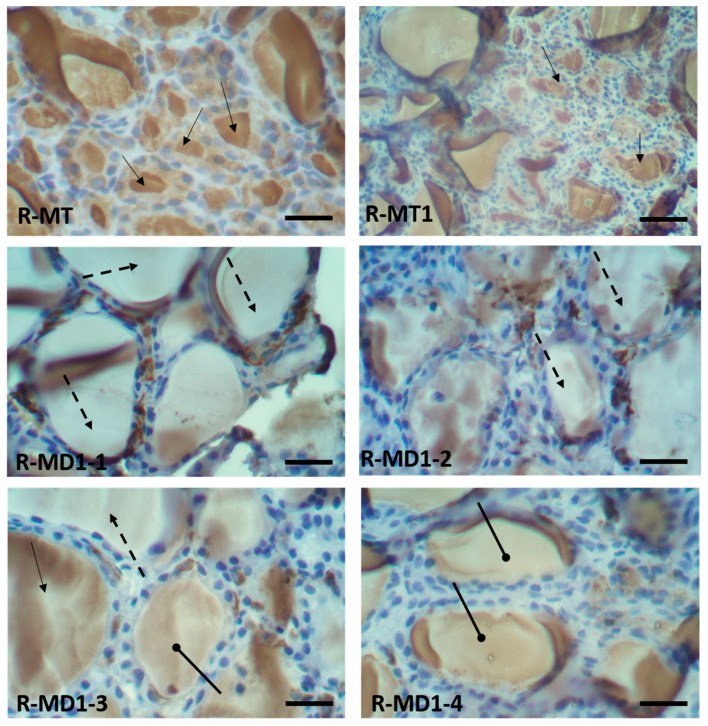
Immunohistochemistry in thyroid sections of 14-day-old rats with specific antibody against thyroglobulin protein. Histological sections of pups in group A (R-MT and R-MT1) and pups in group B (R-MD1-1, R-MD1-2, R-MD1-3 and R-MD1-4).

Immunohistochemistry performed on thyroid sections of 14-day-old rats using a specific antibody against thyroglobulin is presented in Figure 2. Positive immunostaining, indicated by a brown coloration in the colloid, confirmed the presence of thyroglobulin. In the pups in group A (Figure 2. R-MT and R-MT1), strong and widespread immunolabeling was observed, indicating high expression of thyroglobulin.

In contrast, thyroid sections from pups born to mothers in group B throughout gestation and lactation exhibited markedly reduced or absent thyroglobulin staining (Figure 2: R-MD1-1 and R-MD1-2), suggesting impaired thyroid function. Moreover, a noticeably lower intensity of thyroglobulin labeling was observed in other treated pups (Figure 2: R-MD1-3 and R-MD1-4), further supporting the downregulation of thyroglobulin expression following KL exposure.

For thyroglobulin, there was positive immunostaining in the colloid (brown stain). Positive immunostaining (showed the presence of thyroglobulin) was observed in pups in group A (Figure 2: R-MT and R-MT1). Nevertheless, there was negative immunostaining (absence of thyroglobulin) in pups in group B (R-MD1-1 and R-MD1-2). We observed very intense labeling with anti-thyroglobulin protein in pups in group A (Figure 2: R-MT and R-MT1). The labeling of thyroglobulin in the pups in group A was very important. However, in group B pups, there was lower intensity of labeling (Figure 2: R-MD1-3 and R-MD1-4). Original magnification 200X and 400 X. 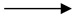: Colloid with brown stain showed positive immunostaining; 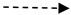: colloid without brown stain showed negative immunostaining; 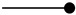: lower intensity of labeling.

### 3.6. Histological Study of Femora

**Figure 3:**
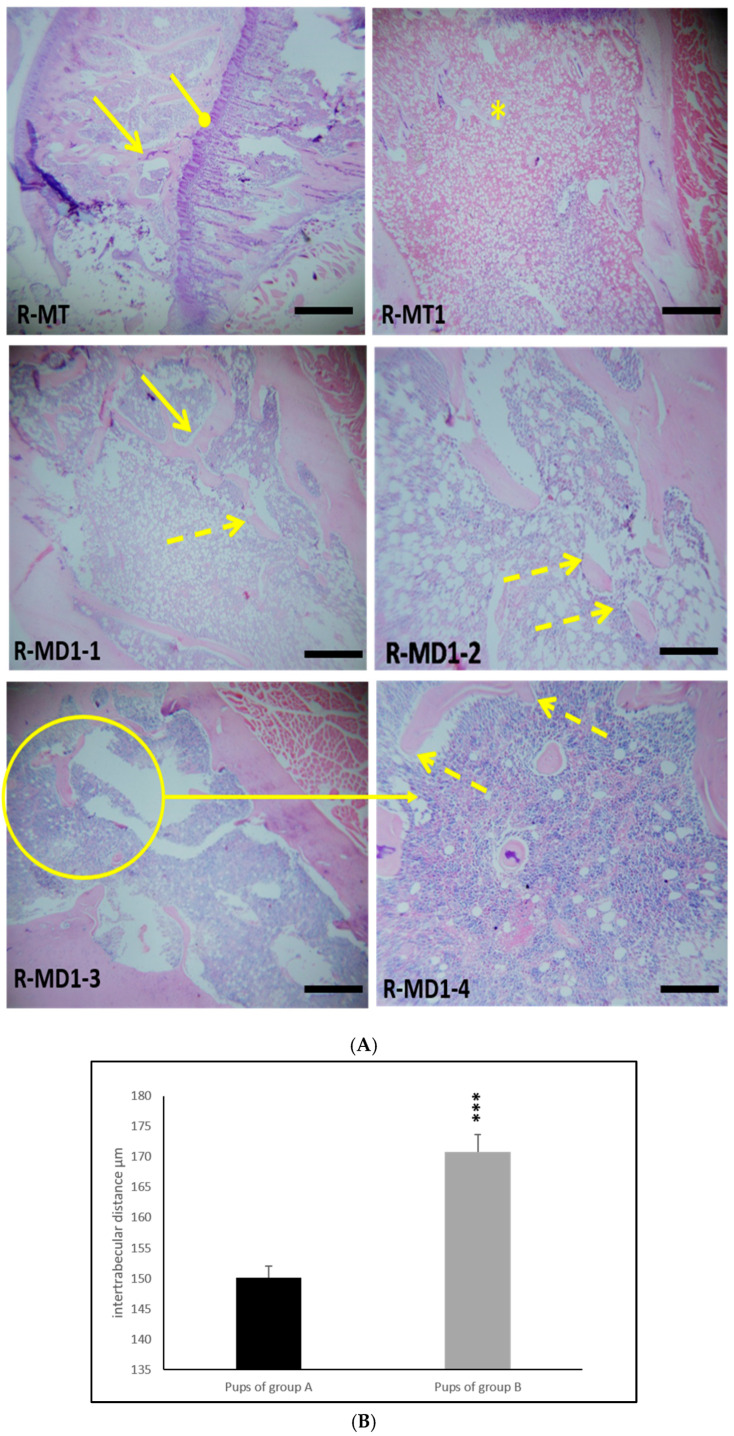
(**A**) H&E-stained sections of distal femora (epiphysis) of 14-day-old rats in group A (R-TM and R-TM1) and pups in group B (R-MD1-1, R-MD1-2, R-MD1-3, and R-MD1-4). Histological study showed a thinning and discontinuity of trabecular bone as well as decreased connectivity of bone trabeculae and disorganization of femur growth plate. (**B**) Histomorphometric analysis of intertrabecular distance in trabecular bone. Values are expressed as means ± SD. Significant differences between pups from group A and group B: *** *p* ≤ 0.001. Original magnification of H&E stain 200X and 400X. 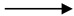: continuous trabecular bone; 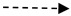: discontinuity of trabecular bone; 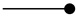: femur growth plate; *: disorganization of femur growth plate.

H&E staining showed normal organization of the growth plate of femora from young rats in group A (Figure 3: R-MT and R-MT1). Hypertrophic and proliferative zones are distinguished in Figure 3 (R-MT). Histological study of femora in young rats in group B indicated a disorganization of the growth plate (Figure 3: R-MD1-1), a disappearance of the nodes (intertrabecular link) and discontinuity of the trabecular spans (Figure 3: R-MD1-2, R-MD1-3, and R-MD1-4). The maturation of chondrocytes to the hypertrophic stage and mineralization of the cartilage had not finished. We can suggest retardation of osteogenesis in young rats in group B.

### 3.7. Histomorphometric Findings in Bone

Histomorphometric analysis revealed significant alterations in the femora of young rats born to dams treated with 1/10 of DL50 of KL (group B) during the prenatal and lactational periods compared to pups in group A (Table 3). Specifically, in pups in group B, exposure to KL resulted in a reduction in osteoid surface (OS/BS), trabecular thickness (Tb. Th), and trabecular bone volume (BV/TV) by 18%, 24%, and 16%, respectively. Additionally, a 13% increase in trabecular separation (Tb.Sp) was observed.

### 3.8. SEM Results

**Figure 4:**
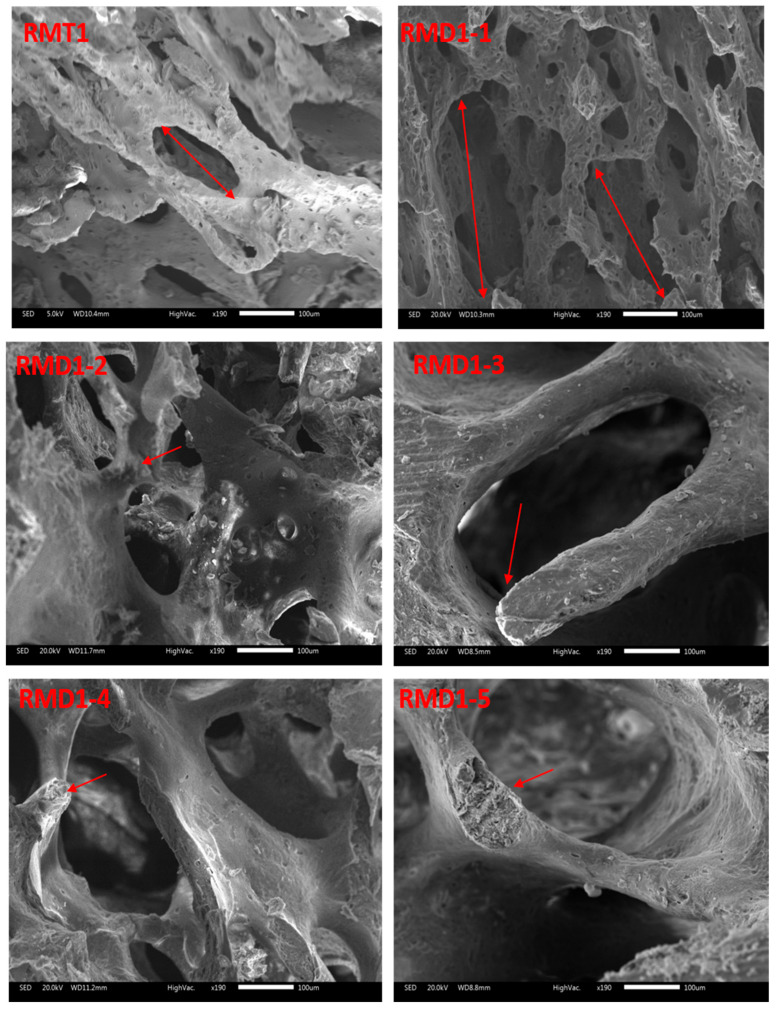
SEM micrographs of femora from pups (14 days old): group A (RMT1) and group B (RMD1-1, RMD1-2, RMD1-3, RMD1-4 and RMD1-5). Decreased connectivity and thinning of bone trabeculae can be observed. Original magnification 20×; 100× and 500×.

Figure 4 displays SEM images of femora of young rats in group A and young rats in group B. The trabecular bone of pups in group A had a homogeneous appearance (Figure 4: RMT1). However, pups in group B displayed separated trabecular bones in the extremity and slimmed trabecular bones in the epiphysis compared to group A (Figure 4: RMD1-1 and RMD1-2).

### 3.9. FTIR Spectral Analysis of Femoral Bone Powder

**Figure 5:**
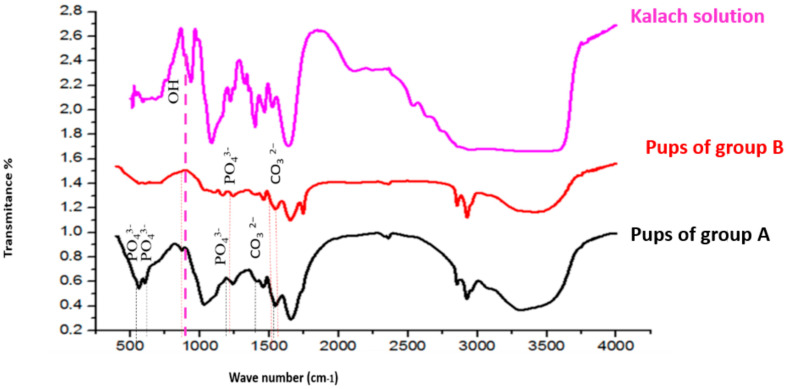
FTIR spectra of bone samples and kalach fluid between 500 and 4000 cm^−1^. The bands in red are significantly different from the spectra of group A and group B.

The FTIR spectra of bone powder collected from the femora of pups in group A and group B are shown in Figure 5. Wave-number shifts observed in the spectra (detailed in Table 4) indicate molecular disturbances induced by KL treatment.

In group A, characteristic vibrations of unstable carbonate ions (CO_3_^2−^) were observed at 1545 cm^−1^ and 1455 cm^−1^. In pups in group B, these bands were shifted to 1539 cm^−1^ and 1458 cm^−1^, respectively. Additionally, the carbonate vibration at 1320 cm^−1^, present in pups in group A, was absent in pups in group B.

Phosphate ion (PO_4_^3−^) vibrations, originally observed at 1242, 1032, 603, and 558 cm^−1^ in group A samples, were shifted in treated rats to 1239, 1035, 608, and 561 cm^−1^, respectively. Furthermore, new vibration bands emerged at 1167 cm^−1^ and 1113 cm^−1^, corresponding to PO_4_^3−^, and at 723 cm^−1^, associated with hydroxyl ions (OH^−^), in group B samples (Figure 5).

### 3.10. X-Ray Diffraction (XRD) Results

**Figure 6:**
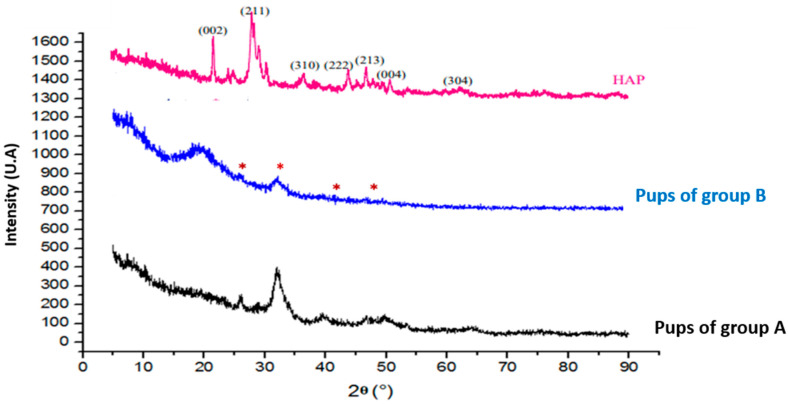
X-ray diffractograms of control and treated rats. *: represent diffraction peak positions in pups of group B.

The X-ray diffraction (XRD) patterns of hydroxyapatite (HAP) and femora of pups in group A were used as reference standards to assess the impact of the herbicide applied in this study (Figure 6). XRD analysis conducted after the prenatal and lactation exposure periods revealed characteristic HAP diffraction peaks at approximately 26°, 32°, 40°, 46.7°, 50°, 53°, and 64° (2θ), corresponding to the (004), (002), (222), (310), (211), (213), and (304) crystal planes, respectively (Figure 6).

Our results showed peak shifts indicative of structural changes. Specifically, exposure to KL led to a decrease in the 2θ values of the (002), (211), and (310) reflections (Table 5). The femoral bone of pups born from group B displayed altered HAP structures. While the general diffraction profile remained similar to that of the control group, noticeable differences were observed. In particular, several peaks in group B were either shifted or absent compared to group A (Figure 6).

Notably, the presence of KL induced a reduction in 2θ values at 26°, 43°, and 49°. Such reductions suggest modifications in the crystal lattice, as lower 2θ values are associated with an increase in crystallographic parameters (a = b, c), ultimately resulting in an expanded unit cell volume in the bone matrix.

### 3.11. In Silico Study Results

The in silico analysis revealed that the interaction of KL with the targeted receptors varied, likely due to differences in structural geometry and the presence of specific heteroatoms. The selected poses reported in the current study were based on the best binding energies together with the root mean square deviation calculation, which was equal to zero, as previously reported [46].

Table 6 summarizes the binding energies, number of conventional hydrogen bonds, closest interacting residues, and distances between KL atoms and receptor residues. Overall, the computational data support a plausible correlation between the molecular interactions of KL and observed biological activity [18,42,46,49].

**Figure 7:**
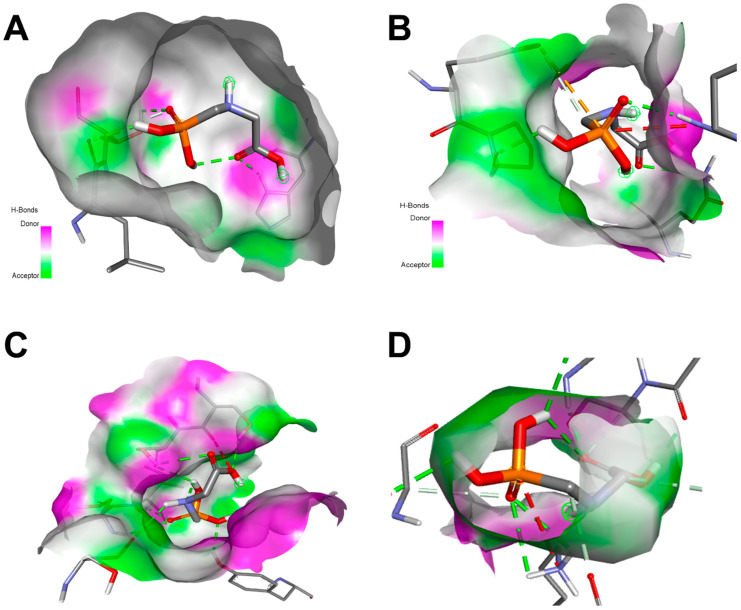
Micrographs of the 3D structure of KL bound to the pocket region of ERα (**A**), ARα (**B**), GH (**C**), and thyroglobulin (**D**). Illustrations of the H-bond interactions within the pocket regions of the targeted receptors.

In particular, KL demonstrated a favorable binding pose with thyroglobulin, forming seven hydrogen bonds and embedding deeply within the binding pocket. The most notable interaction was with the Gly989 residue, at a close distance of 1.944 Å (Table 6). As illustrated in Figure 7, KL bound to the receptors with relatively high negative binding affinities, ranging from −4.4 kcal/mol for ERα to −5.4 kcal/mol for GH, through a network of electrostatic, conventional hydrogen, and carbon–hydrogen bonds.

Among the receptors, GH showed the strongest affinity for KL (−5.4 kcal/mol) and the highest number of hydrogen bonds. KL interacted specifically with residues Ser102, Ile103, Tyr107, and Ser98, and multiple contacts with Ser99. These findings suggest that KL may exert its most significant toxicological effects through its interaction with GH, followed by thyroglobulin (Figure 7).

## 4. Discussion

In the present study, we investigated the effects of KL on the bone tissue and thyroid gland of offspring rats. We analyzed thyroid hormonal status, calcium homeostasis (plasmatic and bone calcium and phosphorus levels) and performed histological evaluations of bone tissue and thyroid glands.

Our study involved continuous perinatal exposure. Previous evidence suggests that the gestational period represents a particularly critical window for thyroid and skeletal development, as key processes such as thyroid gland morphogenesis, hormone synthesis, and bone ossification occur during this time. Lactation may also contribute, but the developing fetus appears to be more susceptible to endocrine disruption. Understanding these critical windows is important for designing future toxicological studies, allowing researchers to focus on gestational exposure periods and for epidemiological investigations aiming to assess potential health risks associated with maternal G exposure.

Metabolic and histological changes were very serious in offspring rats. This study is, to the best of our knowledge, the first to investigate the effects of KL on both the thyroid gland and bone tissue in Wistar rat offspring.

Our results indicate a reduction in body weight observed in 14-day-old rats was accompanied by impaired skeletal growth, as indicated by decreased femur length and weight. These findings are consistent with previous reports, such as Rieg et al. [50]. They showed that perinatal exposure to a commercial formulation of G impaired both growth and body weight gain in rat pups. These findings may also be explained by the reduced food intake observed in female rats subjected to a low-nutrient diet, as reported in our previous study. A decrease in appetite in rats exposed to a GBH often results from a combination of neurological, metabolic, and inflammatory factors [27,51]. The decrease in food intake leads to a reduction in calcium, phosphorus, and protein intake, which can reduce bone formation and slow the growth of young rats [52].

In the present study, we suggests that the thyroid glands of these pups were unable to produce sufficient amounts of TH. Previous studies have demonstrated that G and GBH act as endocrine disruptors in mammals by interfering with hormonal function [3,53]. Moreover, G induces cell death by inhibiting mitochondrial succinate dehydrogenase activity and promotes necrosis through the release of cytosolic acetylated kinase, leading to membrane damage. It has also been shown that G triggers apoptosis via the enzymatic activation of caspases 3 and 7 [54]. In our study, hypothyroidism was primary or secondary. Primary hypothyroidism is a result of a tissue injury observed in the histological analyses of thyroid glands, such as a necrosis (Figure 2). Our results proved that KL led to a disturbance in the histological structure of thyroid follicles, with a decrease in colloid area. It has been shown that KL induces tissue injury and inflammation due to oxidative stress [55]. In addition, a major cause of primary hypothyroidism is iodine deficiency, as iodine is a key precursor in TH biosynthesis. Iodine uptake by the thyroid gland constitutes the initial step in TH production [56]. We observed previously a significant decrease in food intake in female rats exposed to KL compared to a control group [57]. Therefore, we suggest a reduction in iodine nutritional intake. On the other hand, hypothyroidism can be secondary (central) when the pathology is related to the pituitary gland or hypothalamus. The decline in the production of FT4 and FT3 increases in the secretion of thyroid-stimulating hormone (TSH) by the pituitary gland, causing hypertrophy and hyperplasia of the thyroid parenchyma. Secondary hypothyroidism can be the result of interaction with G and GH. Therefore, the in silico study showed that the strongest toxicological effect of KL might be on GH, followed by thyroglobulin. Nevertheless, the binding affinity was higher than with GH.

Taken together, these findings are evidence of the significant effects of KL, particularly the observed biochemical and histological alterations. Consistent with our results, previous studies have emphasized the value of combining in silico and in vivo approaches to assess toxicity and endocrine disruption, including molecular interaction simulations with estrogen and androgen receptors [43,58]. For example, De Souza et al. reported that perinatal exposure to GBH in male rats alters the TSH regulatory set point, possibly through post-translational mechanisms, and disrupts the expression of multiple genes involved in thyroid hormone homeostasis [33]. In our study, the disruption of the calcium–phosphorus balance can be attributed to several pathophysiological mechanisms related to pesticide-induced toxicity in organs regulating mineral metabolism. Although direct evidence linking pesticide exposure to calcium–phosphorus imbalance in pups is limited, research indicates that perinatal GBH exposure can cause liver damage in immature Wistar rats [50], which may indirectly impair mineral regulation given the liver’s role in this process. Similarly, Rieg et al. (2022) [50] demonstrated that perinatal GBH exposure induces calcium influx, oxidative stress, inflammation, and iron accumulation, leading to hepatotoxicity. In addition, the perturbation of calcium–phosphorus balance in pups may also result from the nephrotoxic effects of KL, as both subchronic and acute GBH exposures have been shown to cause nephrotoxicity in adult rats [59]. Calcium is a crucial element for skeletal mineralization, with more than 99% of the body’s calcium stored in bone in the form of hydroxyapatite (HPA) [60]. This mineral not only provides structural strength to the skeleton but also serves as a reservoir, enabling the controlled release of calcium into the bloodstream when required. The regulation of calcium homeostasis relies on a balance between bone turnover, intestinal absorption, and renal reabsorption [60]. The disruption of calcium and phosphorus in bone observed in our study likely results from a combination of hormonal imbalances, direct bone toxicity, kidney dysfunction, and oxidative stress [3,17,59]. It has been reported that hypothyroidism causes general hypo-metabolism [56]. G has been reported to function as an endocrine disruptor, potentially interfering with hormones such as PTH, calcitonin, and Vit D, all of which play key roles in regulating bone metabolism [27,33].

This disruption in calcium homeostasis may impair bone mineralization, leading to weakened bone structure. Histological examination and SEM analysis of bones from offspring exposed to KL revealed disorganization of the growth plate, loss of intertrabecular nodes, and discontinuities in the trabecular network. SEM images corroborated these histological observations. The maturation of chondrocytes to the hypertrophic stage and mineralization of the cartilage had not finished. We can explain this histological observation by hypothyroidism. It is known that hypothyroidism leads to a retardation in 50% of bone formation processes, 40% of bone resorption processes, and bone rarefaction [61]. Hypothyroidism results in growth retardation or cessation, disruptions in endochondral ossification, delayed bone maturation, and persistent short stature [24,62].

Analysis showed disorganization of femoral histomorphometric parameters in pups exposed to KL during the prenatal and lactational periods. This observation may be attributed to an imbalance between osteoclastic and osteoblastic activities, resulting in incomplete bone remodeling, bone rarefaction, and reduced trabecular wall thickness. The biochemical, histological, and histomorphometric findings were consistent and mutually supportive, indicating that KL exposure during prenatal and lactational periods disrupts the bone remodeling process. Since endochondral ossification is a complex process that begins in the fetus and continues through growth, with bone lengthening occurring via growth plate proliferation followed by ossification, any alteration at this stage has significant consequences. In our study, control pups displayed well-developed growth plates with empty cells separated by calcified spans, whereas pups from KL-exposed dams exhibited thinning of trabeculae. These bone alterations can be explained by oxidative stress and inflammation, as GBH has been reported to induce oxidative stress in bone tissue, leading to excessive production of reactive oxygen species (ROS) [17]. These ROS can stimulate osteoclast activity while inhibiting osteoblast function, promoting bone resorption over bone formation. ROS and inflammation may represent potential mechanisms suggested by previous studies [27,51]. Manolagas reported that redox disturbances are involved in the pathogenesis of osteoporosis, and increased ROS was associated with decreased bone formation [63]. Chronic inflammation caused by GBH may further exacerbate bone degradation by altering cytokine signaling involved in bone remodeling [51].

Based on the available literature and our findings, it is scientifically reasonable to propose that the thyroid and bone disturbances observed after gestational and lactational exposure to GBHs may arise from a dual mechanistic pathway. First, direct endocrine disruption through the hypothalamic–pituitary–thyroid (HPT) axis cannot be excluded, as experimental studies have reported alterations in the expression of thyroid-related genes (e.g., NIS, TPO, TG, TSHR, and deiodinases) as well as changes in circulating T3, T4, and TSH levels following GBH exposure, suggesting dysregulation of thyroid hormone synthesis and metabolism in a dose-, age-, and formulation-dependent manner [27,64]. Second, a more consistently supported mechanism involves indirect effects mediated by oxidative stress and inflammatory responses, since GBHs have been shown to induce systemic oxidative stress, increase lipid peroxidation, deplete antioxidant defenses, and trigger inflammatory infiltration in the thyroid gland and other tissues, conditions that are known to impair thyroid follicular function, alter peripheral deiodination, and disturb thyroid hormone transport [3,17,65]. These endocrine perturbations may in turn affect bone homeostasis in offspring, as thyroid hormones are crucial regulators of bone growth, mineralization, and remodeling, and thus disruption of thyroid function during critical developmental windows could contribute to the bone disorders we observed. We hypothesize that both direct interference with thyroid hormone synthesis/metabolism and indirect oxidative stress–inflammation pathways are plausible and may act synergistically to mediate the endocrine and skeletal outcomes associated with perinatal GBH exposure.

Prolonged exposure can alter the crystallinity of bone hydroxyapatite, which can be observed using techniques such as XRD and FTIR, revealing changes in the composition and structure of bone tissue [18,37]. FTIR spectra and XRD analysis in bone of young rats breastfed by mothers treated with KL showed a disturbance at the bone level, caused by the establishment of the herbicide (KL) within the mineral crystalline phase of the bone. This can indicate that there is a direct effect of KL in HAP. As mentioned, G consists of a carboxylic acid group (–COOH). This acid is well known for demineralizing hard tissues by forming more soluble calcium salts, as highlighted by Feitosa et al. [66]. Indeed, an acidic proton should participate in the release and dissolution of calcium from the mineral phase of the bone, leading to an increase in the proportion of demineralized collagen. This effect is explained by the condensation of a carboxylic acid group (–COOH). Furthermore, we believe that all acid monomers react with the mineral phase of the bone (HAP). Gallegos et al. (2016) reported that exposure to GBH during gestation and lactation induces neurobehavioral alterations in rat offspring, including changes in locomotor activity, emotionality, and anxiety-like behavior [34].

Therefore, our results confirm previously reported data about the impact of exposure to chemical compounds on bone dynamics, growth, mass, and microarchitecture [67]. For instance, it is well known that inhibition of each of the targeted proteins (ERα, ARα, GH and thyroglobulin) has direct effects on skeletal development [3,43,61,68]. Nonetheless, our study represents the first investigation into the effect of KL on bone tissue in pups, phosphocalcic equilibrium, and histological parameters in the thyroid gland. Our study revealed that KL significantly affects offspring growth, leading to disturbances in the calcium–phosphorus balance and hypothyroidism. We also demonstrated that perinatal exposure to KL negatively impacts bone mass in suckling rats. This was evidenced by severe bone abnormalities confirmed by histology, SEM, FTIR, XRD, bone histomorphometry, and thyroid gland necrosis. These results confirm that bone destruction in pups rats after exposition to KL can lead to rickets (rachitis) and growth retardation, primarily due to impaired skeletal mineralization.

We acknowledge that direct extrapolation from animal models to humans has inherent limitations. In our study, rats were exposed to controlled doses of GBH, whereas in humans exposure occurs mainly through dietary intake of food residue or occupational handling, usually at lower and more chronic levels. In addition, differences in metabolism, absorption, and placental transfer between rodents and humans may affect toxicokinetics and toxicodynamics [7,8,10,11,12]. The critical developmental windows in rats are not fully comparable to those in humans either, which complicates direct translation. Therefore, while our findings provide valuable mechanistic insights and highlight potential risks for thyroid and skeletal development, they should be interpreted with caution. Further epidemiological investigations and mechanistic studies in human-relevant models are required to better assess the implications for pregnant women and developing fetuses.

## 5. Conclusions

This study offers new insights into the toxicological effects of kalach, a GBH used in Tunisia, on bone tissue in offspring during the perinatal and lactational periods. The findings highlight significant disruptions in mineral homeostasis, including hypercalcemia and decreased phosphorus levels. These disturbances indicate the nephrotoxicity induced by KL and the role of oxidative damage in different organs. Our findings indicate that hypothyroidism results in growth retardation, along with delayed bone development, as evidenced by reduced bone weight and length. Therefore, this hypothyroidism affected skeletal maturation and induced disturbances of endochondral ossification. Such effects may result from the molecular interactions of KL with several targeted receptors, specifically GH and thyroglobulin. Physicochemical studies (FTIR and XRD) further confirmed the harmful impact of KL on the crystalline fraction, composed of hydroxyapatite crystals, of bone tissue of rat offspring. Histological and SEM analyses further confirmed the adverse effects on bone structure, including bone rarefaction, trabecular discontinuities, and necrosis in the thyroid gland. Therefore, this study provides the first detailed exploration of KL’s impact on bone tissue in pups during the perinatal and lactational period, revealing its multifaceted toxicity and significant implications for bone health, systemic mineral metabolism, and the key roles of GH and THs.

## Figures and Tables

**Table 1:** Effect on body weight, femur length and weight of femora in the pups from group A and group B: 1st day of pregnancy until euthanasia of the pups on the 14th day after their birth.

Parameters and Treatments	Body Weight (g)	Food Consumption (g)/day	Femur Length (mm)	Weight of Femora (mg)
Group A	181.75 ± 5.51	90.33 ± 3.55	----	----
Group B	171.91 ± 5.2 *	72.43 ± 2.43 *	----	----
Pups in group A (*n* = 8)	21.27 ± 1.22	---	11.60 ± 0.33	40.36 ± 3.24
Pups in group B (*n* = 8)	17.98 ± 2.03 **	---	10.64 ± 0.51 *	38.58 ± 2.73 **

Group B vs. group A: * *p* ≤ 0.05, **p ≤ 0.01.

**Table 2:** Plasma levels of thyroid hormones (FT4 and FT3), plasma, and bone femur levels of calcium and phosphorus in young rats during the suckling phase of pups in group A and group B.

Parameters and Treatments	Plasma Levels (mg/L)		Plasma Levels (pmol/L)		Bone Levels (mg/L)	
	Calcium (*n* = 8)	Phosphorus (*n* = 8)	FT3 (*n* = 8)	FT4 (*n* = 8)	Calcium (*n* = 8)	Phosphorus (*n* = 8)
**Pups in group A**	2.59 ± 0.39	1.77 ± 0.12	4.18 ± 0.27	26.23 ± 3.35	2.74 ± 0.92	1.91 ± 0.31
**Pups in group B**	3.49 ± 0.44 **	1.64 ± 0.14 *	3.31 ± 0.24 *	20.63 ± 0.24 **	1.84 ± 0.23 **NS**	1.54 ± 0.21 **

Pups in group B vs. group A: * *p* ≤ 0.05, ** *p* ≤ 0.01; NS: non-significant.

**Table 3:** Histomorphometric finding in pups in group A and group B.

Parameters and Treatments	Pups in Group A	Pups in Group B
**BV/TV (%)**	25.28 ± 0.50	21.11 ± 0.61 *
**OS/BS (%)**	1.73 ± 0.22	1.41 ± 0.10 **
**Tb.Sp (µm)**	150.11 ± 1.89	170.8 ± 2.85 **
**Tb.Th (µm)**	69.10 ± 0.77	51.87 ± 1.32 *
**Tb.N**	4.01 ± 0.07	3.18 ± 0.10 NS

Values are expressed as means ± SD. Significant differences between pups in group A and pups in group B: * *p* ≤ 0.05; ** *p* ≤ 0.01, NS: Non-significant.

**Table 4:** Band movements in different groups of rats.

Groups	FTIR (cm^−1^)									
Compound	CO_3_^2−^			PO_4_^3−^						Displaced OH
**Pups in group A**	1320	1455	1539	558	603	1032	----	---	1242	---
**Pups in group B**	---	1458	1545	561	608	1035	1113	1167	1239	723
**KL solution**	1399	1466	1523	589 533 518 512		1083	1163		1247	720

**Table 5:** XRD in different groups of rats.

Groups/Peaks	XRD		
	(002)	(211)	(310)
**HPA**	27	33	41
**Pups in group A**	27	33	41
**Pups in group B**	26	-	43 49

HAP: hydroxyapatite, KL: kalach 360 SL.

**Table 6:** Binding energy, conventional hydrogen bonds and the closest interacting residues of estrogen receptor α, androgen receptor α, and growth hormone receptor.

Target Protein	Binding Energy (kcal/mol)	Interactions with Amino Acid Residues	
		Conventional H-Bonds	Distance to Closest Interacting Residues
**Estrogen Receptor α**	−4.4	4	**Thr347, 2.726 Å; His524, 2.230 Å; Leu346, 2.104 Å; Unk0, 2.494 Å; Thr347, 3.400 Å**
**Androgen Receptor α**	−4.8	3	**Glu681, 4.477 Å; Gln711, 2.992 Å; Arg752, 2.028 Å; Pro682, 2.266 Å; Glu681, 3.671 Å**
**Growth Hormone Receptor**	−5.4	7	**Ser102, 2.423 Å; Ile103, 2.147 Å; Tyr107, 2.187 Å; Ser98, 2.216 Å; Ser99, 2.544 Å; Ser99, 2.732 Å; Ser99, 2.545 Å**
**Thyroglobulin**	−4.7	7	**Lys961, 2.779 Å; Lys961, 2.894 Å; Gly654, 2.051 Å; Gly654, 2.658 Å; Phe632, 2.368 Å; Gly989, 1.944 Å; Ser990, 2.756 Å; Gly634, 3.393 Å; Ser990, 3.213 Å; Val959, 3.738 Å; Phe986, 3.153 Å**

## Data Availability

The data that support the findings of this study are available from the corresponding author upon reasonable request.

## References

[1] Imfeld G., Vuilleumier S. (2012). Measuring the effects of pesticides on bacterial communities in soil: A critical review. Eur. J. Soil. Biol..

[2] Benbrook C.M. (2016). Trends in glyphosate herbicide use in the United States and globally. Environ. Sci. Eur..

[3] Ben Amor M., Hamdaoui L., Daoud S., Ammar M., Louati N., Elleuch A., Badraoui R., Ben Mahmoud L., Ben Amor I., Sellami A. (2025). Impact of sub-chronic exposure to Kalach on male reproductive system and sperm function: In silico modelling and in vivo study in rats. Reprod. Toxicol..

[4] Leoci R., Ruberti M. (2020). Glyphosate in Agriculture: Environmental Persistence and Effects on Animals. A Review. J. Agric. Environ. Int. Dev. JAEID.

[5] Silva V., Montanarella L., Jones A., Fernández-Ugalde O., Mol H.G.J., Ritsema C.J., Geissen V. (2018). Distribution of glyphosate and aminomethylphosphonic acid (AMPA) in agricultural topsoils of the European Union. Sci. Total. Environ..

[6] Dahmeni G., Grünberger O., Chaabane H. (2024). Why and where should glyphosate water contamination be monitored in Tunisia? A review based on Mediterranean situations. Euro Mediterr. J. Environ. Integr..

[7] Roberts D.M., Buckley N.A., Mohamed F., Eddleston M., Goldstein D.A., Mehrsheikh A., Bleeke M.S., Dawson A.H. (2010). A prospective observational study of the clinical toxicology of glyphosate-containing herbicides in adults with acute self-poisoning. Clin. Toxicol..

[8] Fréville M., Henri J., Estienne A., Serra L., Ramé C., Ganier P., Chahnamian M., Froment P., Dupont J. (2023). Determination of the elimination half-life of Glyphosate and its main metabolite, AMPA, in chicken plasma. Toxicol. Lett..

[9] Liu J., Schelar E. (2012). Pesticide Exposure and Child Neurodevelopment: Summary and Implications. Workplace Health Saf..

[10] Daruich J., Zirulnik F., Sofía Gimenez M. (2001). Effect of the Herbicide Glyphosate on Enzymatic Activity in Pregnant Rats and Their Fetuses. Environ. Res..

[11] Mose T., Kjaerstad M.B., Mathiesen L., Nielsen J.B., Edelfors S., Knudsen L.E. (2008). Placental Passage of Benzoic acid, Caffeine, and Glyphosate in an Ex Vivo Human Perfusion System. J. Toxicol. Environ. Health Part A.

[12] Poulsen M.S., Rytting E., Mose T., Knudsen L.E. (2009). Modeling placental transport: Correlation of in vitro BeWo cell permeability and ex vivo human placental perfusion. Toxicol. Vitr..

[13] Bus J.S. (2015). Analysis of Moms Across America report suggesting bioaccumulation of glyphosate in U.S. mother’s breast milk: Implausibility based on inconsistency with available body of glyphosate animal toxicokinetic, human biomonitoring, and physico-chemical data. Regul. Toxicol. Pharmacol..

[14] Gillezeau C., van Gerwen M., Shaffer R.M., Rana I., Zhang L., Sheppard L., Taioli E. (2019). The evidence of human exposure to glyphosate: A review. Environ. Health..

[15] Steinborn A., Alder L., Michalski B., Zomer P., Bendig P., Martinez S.A., Mol H.G.J., Class T.J., Pinheiro N.C. (2016). Determination of Glyphosate Levels in Breast Milk Samples from Germany by LC-MS/MS and GC-MS/MS. J. Agric. Food Chem..

[16] Camiccia M., Candiotto L.Z.P., Gaboardi S.C., Panis C., Kottiwitz L.B.M. (2022). Determination of glyphosate in breast milk of lactating women in a rural area from Paraná state, Brazil. Braz. J. Med. Biol. Res..

[17] Hamdaoui L., Oudadesse H., Lefeuvre B., Mahmoud A., Naifer M., Badraoui R., Ayadi F., Rebai T. (2020). Sub-chronic exposure to Kalach 360 SL, Glyphosate-based Herbicide, induced bone rarefaction in female Wistar rats. Toxicology.

[18] Hamdaoui L., El Feki H., Ben Amor M., Oudadesse H., Badraoui R., Khalil N., Brahmi F., Jilani S., Aloufi B., Ben Amara I. (2025). Physicochemical Exploration and Computational Analysis of Bone After Subchronic Exposure to Kalach 360 SL in Female Wistar Rats. Toxics.

[19] Siddiqui J.A., Partridge N.C. (2016). Physiological Bone Remodeling: Systemic Regulation and Growth Factor Involvement. Physiology.

[20] Florencio-Silva R., Sasso G.R.D.S., Sasso-Cerri E., Simões M.J., Cerri P.S. (2015). Biology of Bone Tissue: Structure, Function, and Factors That Influence Bone Cells. BioMed Res. Int..

[21] Briffa J.F., O’Dowd R., Romano T., Muhlhausler B.S., Moritz K.M., Wlodek M.E. (2019). Reducing Pup Litter Size Alters Early Postnatal Calcium Homeostasis and Programs Adverse Adult Cardiovascular and Bone Health in Male Rats. Nutrients.

[22] Kovacs C.S. (2015). Calcium, phosphorus, and bone metabolism in the fetus and newborn. Early Hum. Dev..

[23] Ballock R.T., Reddi A.H. (1994). Thyroxine is the serum factor that regulates morphogenesis of columnar cartilage from isolated chondrocytes in chemically defined medium. J. Cell Biol..

[24] Harvey C.B., O’SHea P.J., Scott A.J., Robson H., Siebler T., Shalet S.M., Samarut J., Chassande O., Williams G.R. (2002). Molecular Mechanisms of Thyroid Hormone Effects on Bone Growth and Function. Mol. Genet. Metab..

[25] Pearce E.N. (2024). Endocrine Disruptors and Thyroid Health. Endocr. Pract..

[26] De Souza C.A., Kayano A.M., Setúbal S.S., Pontes A.S., Furtado J.L., Kwasniewski F.H., Zaqueo K.D., Soares A.M., Stábeli R.G., Zuliani J.P. (2012). Local and systemic biochemical alterations induced by Bothrops atrox snake venom in mice. J. Venom. Res..

[27] Oliveira J.M., Zenzeluk J., Bargi-Souza P., Szawka R.E., Romano M.A., Romano R.M. (2023). The effects of glyphosate-based herbicide on the hypothalamic-pituitary thyroid axis are tissue-specific and dependent on age exposure. Environ. Pollut..

[28] Badraoui R., Sahnoun Z., Abdelmoula N.B., Hakim A., Fki M., Rebaï T. (2007). May antioxidants status depletion by Tetradifon induce secondary genotoxicity in female Wistar rats via oxidative stress?. Pestic. Biochem. Physiol..

[29] Badraoui R., Abdelmoula N.B., Feki N., Nasr H.B., Rebai T. (2010). Endocrine disruption and ovarian morphometric responses in rats following exposure to tetradifon. Gen. Comp. Endocrinol..

[30] Selli J., Unal D., Mercantepe F., Akaras N., Kabayel R., Unal B., Atilay H. (2016). Protective effects of beta glucan in brain tissues of post-menopausal rats: A histochemical and ultra-structural study. Gynecol. Endocrinol..

[31] Badraoui R., Ben-Nasr H., Bardakçi F., Rebai T. (2020). Pathophysiological impacts of exposure to an endocrine disruptor (tetradifon) on α–amylase and lipase activities associated metabolic disorders. Pestic. Biochem. Physiol..

[32] Cattani D., De Liz Oliveira Cavalli V.L., Heinz Rieg C.E., Domingues J.T., Dal-Cim T., Tasca C.I., Silva F.R.M.B., Zamoner A. (2014). Mechanisms underlying the neurotoxicity induced by glyphosate-based herbicide in immature rat hippocampus: Involvement of glutamate excitotoxicity. Toxicology.

[33] de Souza J.S., Kizys M.M.L., da Conceição R.R., Glebocki G., Romano R.M., Ortiga-Carvalho T.M., Giannocco G., da Silva I.D.C.G., da Silva M.R.D., Romano M.A. (2017). Perinatal exposure to glyphosate-based herbicide alters the thyrotrophic axis and causes thyroid hormone homeostasis imbalance in male rats. Toxicology.

[34] Gallegos C.E., Bartos M., Bras C., Gumilar F., Antonelli M.C., Minetti A. (2016). Exposure to a glyphosate-based herbicide during pregnancy and lactation induces neurobehavioral alterations in rat offspring. NeuroToxicology.

[35] Paganelli A., Gnazzo V., Acosta H., López S.L., Carrasco A.E. (2010). Glyphosate-Based Herbicides Produce Teratogenic Effects on Vertebrates by Impairing Retinoic Acid Signaling. Chem. Res. Toxicol..

[36] Fischbeck K.L., Rasmussen K.M. (1987). Effect of Repeated Reproductive Cycles on Maternal Nutritional Status, Lactational Performance and Litter Growth in Ad Libitum-Fed and Chronically Food-Restricted Rats. J. Nutr..

[37] Mahmoudi A., Ghorbel H., Feki I., Bouallagui Z., Guermazi F., Ayadi L., Sayadi S. (2018). Oleuropein and hydroxytyrosol protect rats’ pups against bisphenol A induced hypothyroidism. Biomed. Pharmacother..

[38] Ghorbel H., Fetoui H., Mahjoubi A., Guermazi F., Zeghal N. (2008). Thiocyanate effects on thyroid function of weaned mice. Comptes. Rendus. Biol..

[39] Strissel K.J., Stancheva Z., Miyoshi H., Perfield J.W., DeFuria J., Jick Z., Greenberg A.S., Obin M.S. (2007). Adipocyte Death, Adipose Tissue Remodeling, and Obesity Complications. Diabetes.

[40] Mabrouk M., Mostafa A.A., Oudadesse H., Mahmoud A.A., El-Gohary M.I. (2014). Effect of ciprofloxacin incorporation in PVA and PVA bioactive glass composite scaffolds. Ceram. Int..

[41] Atwa A., Mostafa M., Song S., Sarhan M. (2021). The Femoral Head Epiphysis of Ovariectomized Rats as A Site for Studies on Osteoporosis: Microstructural Changes Evaluations. Egypt. Acad. J. Biol. Sci. Histol. Histochem..

[42] Allouche M., Ishak S., Ben Ali M., Hedfi A., Almalki M., Karachle P.K., Harrath A.H., Abu-Zied R.H., Badraoui R., Boufahja F. (2022). Molecular interactions of polyvinyl chloride microplastics and beta-blockers (Diltiazem and Bisoprolol) and their effects on marine meiofauna: Combined in vivo and modeling study. J. Hazard. Mater..

[43] Akacha A., Badraoui R., Rebai T., Zourgui L. (2022). Effect of Opuntia ficus indica extract on methotrexate-induced testicular injury: A biochemical, docking and histological study. J. Biomol. Struct. Dyn..

[44] Badraoui R., Adnan M., Bardakci F., Alreshidi M.M. (2021). Chloroquine and Hydroxychloroquine Interact Differently with ACE2 Domains Reported to Bind with the Coronavirus Spike Protein: Mediation by ACE2 Polymorphism. Molecules.

[45] Badraoui R., Saeed M., Bouali N., Hamadou W.S., Elkahoui S., Alam M.J., Siddiqui A.J., Adnan M., Saoudi M., Rebai T. (2022). Expression Profiling of Selected Immune Genes and Trabecular Microarchitecture in Breast Cancer Skeletal Metastases Model: Effect of α–Tocopherol Acetate Supplementation. Calcif. Tissue. Int..

[46] Zammel N., Saeed M., Bouali N., Elkahoui S., Alam J.M., Rebai T., Kausar M.A., Adnan M., Siddiqui A.J., Badraoui R. (2021). Antioxidant and Anti-Inflammatory Effects of Zingiber officinale roscoe and Allium subhirsutum: In Silico, Biochemical and Histological Study. Foods.

[47] Amri N., Rahmouni F., Chokri M.A., Rebai T., Badraoui R. (2017). Histological and biochemical biomarkers analysis reveal strong toxicological impacts of pollution in hybrid sparrow (*Passer domesticus* × *Passer hispaniolensis*) in southern Tunisia. Environ. Sci. Pollut. Res..

[48] Badraoui R., Abdelmoula N.B., Sahnoun Z., Fakhfakh Z., Rebai T. (2007). Effect of subchronic exposure to tetradifon on bone remodelling and metabolism in female rat. Comptes. Rendus. Biol..

[49] Hchicha K., Korb M., Badraoui R., Naïli H. (2021). A novel sulfate-bridged binuclear copper(II) complex: Structure, optical, ADMET and in vivo approach in a murine model of bone metastasis. New J. Chem..

[50] Rieg C.E.H., Cattani D., Naspolini N.F., Cenci V.H., De Liz Oliveira Cavalli V.L., Jacques A.V., Nascimento M.V.P.D.S., Dalmarco E.M., De Moraes A.C.R., Santos-Silva M.C. (2022). Perinatal exposure to a glyphosate pesticide formulation induces offspring liver damage. Toxicol. Appl. Pharmacol..

[51] Bartholomew S.K., Winslow W., Sharma R., Pathak K.V., Tallino S., Judd J.M., Leon H., Turk J., Pirrotte P., Velazquez R. (2024). Glyphosate exposure exacerbates neuroinflammation and Alzheimer’s disease-like pathology despite a 6-month recovery period in mice. J. Neuroinflamm..

[52] Howe J.C., Beecher G.R. (1983). Dietary Protein and Phosphorus: Effect on Calcium and Phosphorus Metabolism in Bone, Blood and Muscle of the Rat. J. Nutr..

[53] Gasnier C., Dumont C., Benachour N., Clair E., Chagnon M.C., Séralini G.E. (2009). Glyphosate-based herbicides are toxic and endocrine disruptors in human cell lines. Toxicology.

[54] Benachour N., Séralini G.E. (2009). Glyphosate Formulations Induce Apoptosis and Necrosis in Human Umbilical, Embryonic, and Placental Cells. Chem. Res. Toxicol..

[55] Hamdaoui L., Naifar M., Rahmouni F., Ayadi F., Rebai T. (2019). Sub-chronic exposure to Kalach 360 SL–induced damage in rats’ liver and hematological system. Environ. Sci. Pollut. Res..

[56] Delitala A.P., Scuteri A., Doria C. (2020). Thyroid Hormone Diseases and Osteoporosis. J. Clin. Med..

[57] Hamdaoui L., Naifar M., Mzid M., Ben Salem M., Chtourou A., Makni-Ayadi F., Sahnoun Z., Rebai T. (2016). Nephrotoxicity of Kalach 360 SL: Biochemical and histopathological findings. Toxicol. Mech. Methods..

[58] Jeong J., Kim H., Choi J. (2019). In Silico Molecular Docking and In Vivo Validation with Caenorhabditis elegans to Discover Molecular Initiating Events in Adverse Outcome Pathway Framework: Case Study on Endocrine-Disrupting Chemicals with Estrogen and Androgen Receptors. Int. J. Mol. Sci..

[59] Wunnapuk K., Gobe G., Endre Z., Peake P., Grice J.E., Roberts M.S., Buckley N.A., Liu X. (2014). Use of a glyphosate-based herbicide-induced nephrotoxicity model to investigate a panel of kidney injury biomarkers. Toxicol. Lett..

[60] Matikainen N., Pekkarinen T., Ryhänen E.M., Schalin-Jäntti C. (2021). Physiology of Calcium Homeostasis. Endocrinol. Metab. Clin. N. Am..

[61] Yu W., Wu N. (2020). Primary hypothyroidism with pituitary hyperplasia characterized by hypogonadotropic hypogonadism: A case report and review of the literature. Ann. Palliat. Med..

[62] Duncan Bassett J.H., Williams G.R. (2003). The molecular actions of thyroid hormone in bone. Trends. Endocrinol. Metab..

[63] Manolagas S.C. (2010). From Estrogen-Centric to Aging and Oxidative Stress: A Revised Perspective of the Pathogenesis of Osteoporosis. Endocr. Rev..

[64] Phusate V.T. (2021). Changes in Male Rat Thyroid Gland Exposed to Roundup^®^ and Gramoxone^®^. Indian J. Sci. Technol..

[65] El-Shenawy N.S. (2009). Oxidative stress responses of rats exposed to Roundup and its active ingredient glyphosate. Environ. Toxicol. Pharmacol..

[66] Feitosa V.P., Sauro S., Ogliari F.A., Stansbury J.W., Carpenter G.H., Watson T.F., Sinhoreti M.A., Correr A.B. (2014). The role of spacer carbon chain in acidic functional monomers on the physicochemical properties of self-etch dental adhesives. J. Dent..

[67] Mzid M., Badraoui R., Khedir S.B., Sahnoun Z., Rebai T. (2017). Protective effect of ethanolic extract of *Urtica urens* L. against the toxicity of imidacloprid on bone remodeling in rats and antioxidant activities. Biomed. Pharmacother..

[68] Cannarella R., Barbagallo F., Condorelli R.A., Aversa A., La Vignera S., Calogero A.E. (2019). Osteoporosis from an Endocrine Perspective: The Role of Hormonal Changes in the Elderly. J. Clin. Med..

